# The First Fifty ABO Blood Group Incompatible Kidney Transplantations: The Rotterdam Experience

**DOI:** 10.1155/2014/913902

**Published:** 2014-02-06

**Authors:** Madelon van Agteren, Willem Weimar, Annelies E. de Weerd, Peter A. W. te Boekhorst, Jan N. M. Ijzermans, Jaqueline van de Wetering, Michiel G. H. Betjes

**Affiliations:** ^1^Department of Internal Medicine, Division of Nephrology & Transplantation, Erasmus Medical Center, D414, P.O. Box 2040, 3000 CA Rotterdam, The Netherlands; ^2^Department of Hematology, Erasmus Medical Center, P.O. Box 2040, 3000 CA Rotterdam, The Netherlands; ^3^Department of Surgery, Erasmus Medical Center, P.O. Box 2040, 3000 CA Rotterdam, The Netherlands

## Abstract

This study describes the single center experience and long-term results of ABOi kidney transplantation using a pretransplantation protocol involving immunoadsorption combined with rituximab, intravenous immunoglobulins, and triple immune suppression. Fifty patients received an ABOi kidney transplant in the period from 2006 to 2012 with a follow-up of at least one year. Eleven antibody mediated rejections were noted of which 5 were mixed antibody and cellular mediated rejections. Nine cellular mediated rejections were recorded. Two grafts were lost due to rejection in the first year. One-year graft survival of the ABOi grafts was comparable to 100 matched ABO compatible renal grafts, 96% versus 99%. At 5-year follow-up, the graft survival was 90% in the ABOi versus 97% in the control group. Posttransplantation immunoadsorption was not an essential part of the protocol and no association was found between antibody titers and subsequent graft rejection. Steroids could be withdrawn safely 3 months after transplantation. Adverse events specifically related to the ABOi protocol were not observed. The currently used ABOi protocol shows good short and midterm results despite a high rate of antibody mediated rejections in the first years after the start of the program.

## 1. Introduction

Matching for the antigens of the human ABO blood group system is necessary when foreign cells or organs are considered for donation. If not matched properly, the circulating anti-A and/or anti-B blood group antibodies of the recipient will bind to the antigenic moieties of the cell surface-bound A and B blood group molecules within the kidney transplant [1]. The antibodies attached will activate the complement system leading to local cell damage and eventually cell and organ destruction [2]. Therefore, ABO-incompatible (ABOi) kidney transplantation carries a high risk for acute and irreversible antibody mediated rejection and cannot be performed without pretreatment of the recipient [3, 4].

Pretreatment of the recipient is aimed at substantial lowering of the concentration of circulation antibodies before transplantation and reducing the subsequent production of these antibodies. To this end, a number of protocols have been developed that originally included plasmapheresis for antibody removal and splenectomy for permanent reduction of antibody production. In most protocols, high dose intravenous immunoglobulins were also perioperatively given as they exert a pleiotropic immune suppressive effect, particularly in the case of antibody mediated immune diseases. However, in recent years new protocols have been developed based on the use of the B cell depleting antibody rituximab and the availability of an immunoadsorption column that specifically binds anti-A or anti-B antibodies. This column is able to clear efficiently these antibodies from the plasma, thereby obviating the need for plasma exchange. The Swedish ABOi kidney transplantation protocol was among the first that successfully combined these new treatment modalities into a highly effective pretreatment protocol [5]. Published data have shown remarkable good short- and long-term acceptance and function of the ABOi transplanted kidneys using this protocol [6, 7].

In our transplantation center in The Netherlands, we adopted the Swedish protocol and started the ABOi transplantation program in 2006. Over the years, we have left out parts of this protocol and the use of steroids was stopped after 3 months in the posttransplantation period, similar to our standard immune suppressive protocol for ABOc patients.

The long-term results of the first 50 ABOi kidney transplants in a period of 5 years are now reported in detail and safety and long-term results are in accordance with other reports.

However, early antibody mediated rejections were observed more frequently than previously described although in the majority timely treatment was effective. Postoperative removal of antibodies and continuation of prednisone beyond three months after transplantation did not appear to be essential for the success of the program.

## 2. Patients and Protocol

Living ABOi kidney donor-recipient combinations were evaluated for the ABOi procedure after routine pretransplantation screening. Patients with titers of IgM and IgG antibodies against blood group A or B below or equal to 1 : 128 were considered eligible for ABOi kidney transplantation. At first, only O recipients and AB donors were included. The remaining ABOi couples participated in the Dutch national kidney exchange program, because of their good chance to find suitable donors. The protocol described by Tydén et al. [5] was followed with the exception that plasma for immunoadsorption was generated from the blood by a plasma separating dialyzer and not by centrifugation. The immunoadsorption was performed using a specific adsorption column for anti-A or anti-B antibodies (Glycorex Transplantation AB, Lund, Sweden). If needed, the routine hemodialysis session was combined with the immunoadsorption procedure. Such a simultaneous session was performed without specific problems and with similar adequacy as immunoadsorption alone. All patients were given a single dose rituximab (375 mg/m^2^) one month prior to kidney transplantation. Two weeks before transplantation, mycophenolic acid (1000 mg bid), tacrolimus twice daily (target through level 10–15 mg/L), and prednisone 20 mg once daily were given. The immunoadsorption procedure was performed daily before the transplantation. The number of sessions was dependent on the height of the titer of antidonor blood group antibodies and the rebound after every session. Therefore, the number of pretransplantation immunoadsorptions varied with a median of 4 sessions (range from 0 to 7). During the procedure, 6 liters of plasma was passed over the column and antibody titers were assessed before and after the procedure. The kidney transplantation was performed the day after the last immunoadsorption but only if the post-adsorption IgM and IgG antibody titers were below 1 : 8. In 4 patients no immunoadsorption was performed as the anti-ABO titers were already <1 : 8. Following the original protocol, we performed 3 immunoadsorptions at days 1, 4, and 7 postoperatively in the first 25 patients. The day before kidney transplantation, after the last immunoadsorption, 0.5 gram/kg IVIG was given. In all patients, prednisone was stopped 3 months after transplantation and tacrolimus and mycophenolic acid continued, following our standard posttransplantation treatment protocol. No T cell depleting agent was used for induction therapy.

The percentage of CMV seropositive patients was 66% and routinely all our transplantation patients, except for CMV−/− combinations, received prophylaxis with valganciclovir during the first 6 months after kidney transplantation. Routine kidney biopsies were performed in the first years of our ABOi kidney transplantation program but this policy was abandoned, as it did not contribute to clinical decision-making (see Section 3).

A kidney biopsy by indication was performed if rejection was suspected on the grounds of an unexpected halt in improvement or worsening of kidney function. Histological confirmation of rejection was obtained by following the Banff criteria 07 for acute cellular and antibody mediated rejection (AMR). Acute AMR was treated with extra immunoadsorptions, high dose steroids (1000 mg prednisolone/day for 3 days), and IVIG (1 gram/kg). In case of unresponsiveness, T cell depleting therapy was given according to our local protocol. The first 25 patients received postoperative immunoadsorptions per protocol and were routinely monitored for isoagglutinin titers after transplantation. As the isoagglutinin titers remained low and no rebound occurred, we abandoned the policy of routine posttransplantation immunoadsorptions.

For the purpose of comparison, 2 cases of ABO compatible kidney transplantation were selected from the same period and matched for age and number of HLA mismatches for every case of ABOi kidney transplantation. The posttransplantation immune suppressive medication protocol was similar to the ABOi protocol except for induction therapy with basiliximab in all patients from 2007 onwards.

### 2.1. Determination of Antibody Titers

Patient serum was tested for the presence of IgM and IgG antibodies against the ABO-blood group antigens of the donor using the red blood cells (RBC) of the donor. Hemagglutination was assessed fully automated using the ORTHO BioVue system column agglutination technology. Test RBC, with or without serum, were placed in the chamber above the 6-microcolumn cassette preloaded with diluent and/or reagent and glass beads. Upon centrifugation, RBC are forced through the bead column where agglutinated cells are trapped, while unagglutinated RBC travel to the bottom of the column forming a discrete pellet. The isohemagglutinin titer is determined by the highest dilution of patient serum that still results in donor RBC agglutination.

### 2.2. Statistical Analysis

The SPSS software version 18.0 was used for all statistical tests. Descriptive statistics were used to summarize baseline characteristics. Distribution of data was tested using the Kolmogorov-Smirnov test. Continuous variables with a normal distribution are presented as means (SD) and compared using parametric *t*-tests. Skewed distributed continuous variables are presented as medians and compared using the nonparametric Mann-Whitney *U* test. Categorical variables are presented as numbers and/or percentages. For selected comparison between two group proportions, the chi-square test was used. Survival percentages were analyzed with the Kaplan-Meier method. A *P* value of less than 0.05 was considered significant; all probabilities were 2-tailed.

## 3. Results

### 3.1. Patient's Characteristics

From the start of the ABOi program in 2006 until March 2012, a total of 50 patients received an ABOi kidney transplant from a living donor and clinical follow-up was at least one year with a median follow-up of 38 months. The clinical and demographical patient characteristics are shown in Table 1. The majority of ABOi donor-recipient combinations were from an A and/or B positive donor to an O positive recipient (Table 1). This selection is caused by our national living kidney donor exchange program, which can accommodate an appropriate ABO compatible match for most cases except the O recipients and A and B donors.

### 3.2. Kidney Allograft Survival of ABOi Transplantations

**Figure 1:**
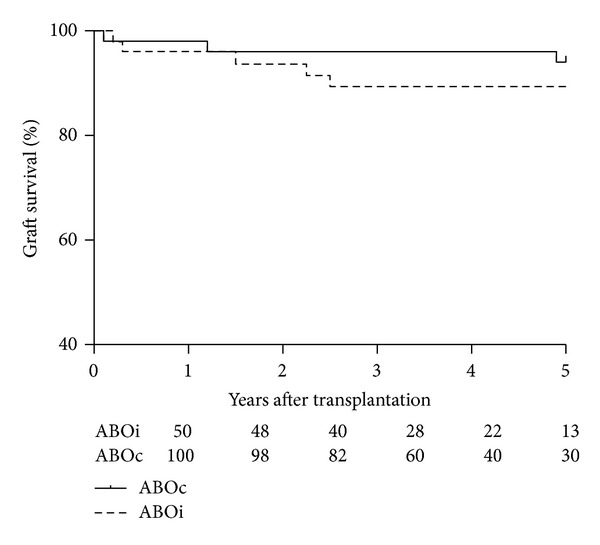
Renal allograft survival censored for death in the blood group ABO incompatible (*n* = 50) and matched ABO compatible (*n* = 100) transplantations. Numbers of patients in study at every year after transplantation are shown below the *x*-axis.

The majority of ABOi patients showed an uncomplicated clinical course and graft survival censored for death at 1 year, 3 years, and 5 years was 96%, 90%, and 90% compared to, respectively, 98%, 96%, and 96% in the ABOc group (Figure 1). ABOi graft survival was slightly worse but not statistically different from the ABO compatible control group (log rank analysis *P* = 0.43). Two patients in the ABOi group died during follow-up; one committed suicide and one patient died because of abdominal sepsis due to an incarcerated hernia cicatricalis. Serum creatinine concentrations at 1 and 3 years follow-up were significantly higher (*P* < 0.05) in the ABOi group (median 137 *μ*mol/L and 154 *μ*mol/L) compared to the ABOc group (median 114 *μ*mol/L and 123 *μ*mol/L). This difference in graft function was largely attributable to the decreased graft function in ABOi patients after their AMR had resolved. The median serum creatinine concentration in the ABOi patient group without AMR was 135 *μ*mol/L at 1 year and 124 *μ*mol/L at 3 years follow-up. For ABOi patients with early AMR, these values were 172 *μ*mol/L and 172 *μ*mol/L, respectively. Two patients had an exceptionally poor renal function at 3 months after transplantation. One case was caused by a surgical problem leading to severe problems with renal blood supply and subsequent partial renal infarction and the other case was due to an AMR resistant to treatment. Of note is one patient that presented 5 months after transplantation with an urosepsis with *Serratia* species and a very severe acute AMR necessitating transplantectomy. Anti-A titers were sharply risen (IgM > 5000 and IgG 512).

### 3.3. Kidney Allograft Rejection of ABOi Transplantations

In eleven patients, acute AMR was observed, all within the first week after transplantation except for the case of late acute AMR described above. Donor specific antibodies against HLA were only found in 2 patients, both having anti-HLA-DQ7 antibodies with a MFI of 20.000 in the single antigen beads Luminex assay. The C4d staining was positive in 8 biopsies, weak to focally positive in 2, and negative in 1 kidney biopsy. Five of these early rejections were categorized as AMR at the time of biopsy and responded to treatment with IVIG and steroids. In 5 cases, the renal biopsy showed a mixed type rejection with evidence for the coexistence of both cellular and AMR. T cell depleting therapy was used in case of an inadequate response to IVIG and steroids. In one of these patients, graft loss occurred 6 months after transplantation because of treatment resistant AMR. One patient had an acute cellular rejection at 7 months when his tacrolimus through level was inadvertently low.

Routine allograft kidney biopsies at day 7 after kidney transplantation showed C4d positive staining in 8 out of 19 (42%) patients but without other positive criteria to support the diagnosis of AMR.

We could not find any significant relation between antibody titers postoperatively and subsequent AMR, as all titers remained low after transplantation (<1 : 8, data not shown). Because of this observation, we stopped the posttransplantation immunoadsorptions after the first 25 patients. This change in protocol did not alter the frequency of AMR and, in fact, most AMR episodes were observed in the patients that received posttransplantation immunoadsorptions (9 out of 11 patients with AMR). In the group of ABOi patients with an early AMR episode, only one patient was diagnosed with transplant glomerulopathy seven years after transplantation.

### 3.4. Adverse Events

All adverse events, possibly related to a change in immune suppression, notably the use of rituximab and IVIG, were recorded. The posttransplantation viral infections in the ABOi group were caused by cytomegalovirus (*n* = 2), BK virus (*n* = 3), and herpes zoster virus (*n* = 3). In addition, 1 case of *Pneumocystis carinii* pneumonia was recorded. The frequency of these infections is in the range of our ABO compatible program and cannot be specifically related to the use of rituximab or IVIG.

## 4. Discussion

Newly developed desensitization protocols have greatly facilitated the development of ABOi kidney transplantation programs in many countries. Similar to other groups, we have successfully applied the Swedish desensitization protocol for more than 5 years and did not observe any significant side effects. By combining the immunoadsorption procedure with hemodialysis, it was also possible to reduce the total number of procedures. This is not only time-effective but also less burdensome for dialysis patients.

In accordance with a number of other studies [8–11], the graft survival of ABOi kidney transplants was not significantly different from matched ABOc kidney transplants. However, it should be noted that the median serum creatinine concentration in the ABOi group was higher at follow-up compared to the ABOc group. This was due to the subgroup of ABOi patients who experienced an AMR. Although the rejection episodes could be treated effectively in most of these patients, the graft function was permanently negatively affected. However, progressive loss of graft function because of ongoing AMR was not observed and overall 5 years graft survival was therefore not significantly lowered compared to the ABOc group. However, the median follow-up of 3 years is relatively short and close monitoring of the ABOi graft results is mandatory. Most cases of AMR occurred within the first 25 patients (9 out of 11 patients with AMR) included in the program and were related to ABO incompatibility, as we could detect DSA only in 2 cases. In spite of a thorough analysis of our procedure, we cannot explain the high frequency of AMR at the start of our ABOi program with a subsequent decrease. The frequency of AMR in study is in contrast to publications by other groups using an identical protocol [8, 9, 12] but a similar or higher rate of rejection has been described by others using different pretransplantation desensitization procedures [13–15]. This highly variable incidence of AMR between the published case series is difficult to explain. Although different definitions of acute rejection may play a role, our definition of a rise in serum creatinine concentration combined with biopsy proven AMR did not leave any uncertainty about the diagnosis. Others have found an increased frequency of AMR in blood group O recipients [16], which may have negatively influenced our results, as the majority of patients in our study carried the blood group O serotype. However, Tydén et al. had a similar high percentage of these patients in their ABOi program but still had a remarkable low incidence of AMR [7]. Therefore, the relevance of the O blood group in the recipient remains an open question.

In agreement with the experience of others [17, 18], we used an upper limit for anti-ABO IgG antibody titers of 1 : 256 to allow patients to enter our program. Notably, the determination of antidonor blood type antibody titers is susceptible to subjective interpretation of the semiquantitative test result and the same serum sample may give very different results when independently tested in different laboratories [19]. This large variation in determination of titers precludes any sound comparison between the results from different centers and may partly explain the differences in the incidence of AMR. In our protocol, we excluded patients with high anti-ABO blood group titers, as it was very difficult to reach a titer of <1 : 8 in these cases. However, it is not known what constitutes a save threshold for the pretransplant antibody titer. Again, the lack of a uniform and reliable test for measurement of antibody titers limits analysis of the combined published data.

We did not observe a relation between posttransplantation anti-A and B titers and rejection and all posttransplantation titers remained low. Therefore, we removed the posttransplantation immunoadsorptions from the protocol. As stated before, the frequency of AMR actually was lower in the 25 patients who did not receive the postoperative immunoadsorptions. Other groups have also reported on the results of ABO-incompatible transplantation without posttransplantation plasmapheresis or immunoadsorption, confirming that such a strategy can indeed be followed without a significant increase in the incidence of AMR [11, 14, 15, 20].

The combined elements of the current ABOi protocol have been chosen on a rationale based on the proposed mechanism of action of each drug. For instance, pretreatment with rituximab depletes the circulating B cell population. This may be important in the prevention of increased anti-A and anti-B antibody synthesis after ABOi kidney transplantation as rituximab treatment diminishes de novo humoral immune responses. However, a recent report showed that a rituximab-free ABOi protocol yields similar excellent short- and long-term results after kidney transplantation [21]. This is of considerable interest as rituximab treatment is not only costly but also associated with increased frequencies of viral infections including BK and polyoma viral infections [8, 22, 23].

Given these results, it seems that a simplified protocol with immunoadsorption and immune suppressive drugs in the pretransplantation period is equally effective. The need for IVIG in the ABOi protocol remains to be established as this is not a standard procedure in some other ABO-incompatible kidney transplantation protocols [8, 15, 20, 24].

After transplantation, we routinely stopped prednisone and continued with tacrolimus and MMF. Others found that this policy may lead to an increase in the incidence of acute rejection and is associated with an decreased graft survival [25]. However, prednisone could be safely stopped at 3 months after ABOi kidney transplantation in our group of patients.

In conclusion, the “Swedish protocol” with immunoadsorption, IVIG, and rituximab, added to a standard triple immune suppressive regimen, has been used successfully for more than 5 years in our transplantation center despite an initial high frequency of AMR. It allows for ABOi kidney transplantation with good long-term graft survival comparable to ABO compatible kidney transplantation. Important questions, such as the upper level of anti-A and anti-B that can be accepted before kidney transplantation and the optimal preconditioning treatment, remain to be answered. The ABOi treatment protocol is of value for those patients that cannot be matched to a donor kidney because of blood group incompatibility. Although our center is involved in an effective national donor kidney exchange program, the O positive recipients benefit in particular from the ABOi protocol as they have the lowest chance of success in the kidney exchange program [26, 27].

## Figures and Tables

**Table 1:** Clinical and demographic characteristics of patients receiving a blood type ABO-incompatible (ABOi) kidney transplant and matched ABO-compatible (ABOc) controls.

	ABOi	ABOc	*P* value
Number of patients	50	100	
Age recipient (median and range)	54 years (22–75)	55 years (19–77)	n.s.*
Age donor (median and range)	50 years (26–75)	55.5 years (23–70)	n.s.
Donor male : female ratio	27 : 23	58 : 42	
Recipient male : female ratio	32 : 18	63 : 37	
Previous transplantation (%)	18%	13%	n.s.
Previous pregnancy (%)	30%	34%	n.s.
Previous blood transfusion (%)	20%	24%	n.s.
Preemptive transplantation	10 (20%)	10 (10%)	n.s.
Number HLA MM (median)	4	4	n.s.
ABO blood group recipient (number and % of total)	O 34 (68%)	O 39 (39%)	
	A 8 (16%)	A 45 (45%)	
	B 8 (16%)	B 13 (13%)	
	AB 0 (0%)	AB 3 (3%)	
Percentage of patients with panel reactive antibodies >4%**	10%	4%	n.s.

*n.s.: not significant (*P* value > 0.05), **most recent percentage of panel reactive antibodies positivity obtained before kidney transplantation. A PRA of <5% was considered as negative by the reference laboratory (Leiden, The Netherlands).

## Abbreviations

ABOi: ABO blood group incompatible
ABOc: ABO blood group compatible
PRA: Panel reactive antibodies
AMR: Antibody-mediated rejection.
